# Amplification of interlimb reflexes evoked by stimulating the hand simultaneously with conditioning from the foot during locomotion

**DOI:** 10.1186/1471-2202-14-28

**Published:** 2013-03-13

**Authors:** Tsuyoshi Nakajima, Trevor Barss, Taryn Klarner, Tomoyoshi Komiyama, E Paul Zehr

**Affiliations:** 1 Department of Integrative Physiology, Kyorin University School of Medicine, 6-20-2 Shinkawa, Mitaka, Japan; 2 Rehabilitation Neuroscience Laboratory, University of Victoria, PO Box 3010 STN CSC, V8W 3P1, Victoria, BC, Canada; 3 Department of Health and Sports Sciences, Faculty of Education, Chiba University, Chiba, Japan; 4 Human Discovery Science, International Collaboration on Repair Discoveries (ICORD), Vancouver, BC, Canada; 5 Centre for Biomedical Research, University of Victoria, Victoria, BC, Canada; 6 Division of Medical Sciences, University of Victoria, Victoria, BC, Canada; 7 School of Exercise Science, Physical, and Health Education, University of Victoria, Victoria, BC, Canada

**Keywords:** Reflex, Interlimb, Propriospinal, Central pattern generator, Afferent feedback, Rehabilitation, Walking, Cycling, Arm swing

## Abstract

**Background:**

Widespread interlimb reflexes evoked in leg muscles by cutaneous stimulation of the hand are phase-modulated and behaviorally relevant to produce functional changes in ankle trajectory during walking. These reflexes are complementary to the segmental responses evoked by stimulation at the ankle. Despite differences in the expression of reflex amplitude based upon site of nerve stimulation, there are some common features as well, suggesting the possibility of shared interneuronal pathways. Currently little is known about integration or shared reflex systems from interlimb cutaneous networks during human locomotion. Here we investigated convergent reflex effects following cutaneous stimulation of the hand and foot during arm and leg cycling (AL) by using spatial facilitation. Participants performed AL cycling and static activation of the target muscle knee extensor vastus lateralis (VL) in 3 different randomly ordered nerve stimulation conditions: 1) superficial radial nerve (SR; input from hand); 2) superficial peroneal nerve (SP; input from foot); and, 3) combined stimulation (SR + SP). Stimuli were applied around the onset of rhythmic EMG bursts in VL corresponding to the onset of the power or leg extension phase.

**Results:**

During AL cycling, small inhibitory (~80 ms) and large facilitatory reflexes (~100 ~ 150 ms) were seen in VL. The amplitudes of the facilitatory responses with SR + SP stimulation were significantly larger than those for SP or SR stimulation alone. The facilitation was also significantly larger than the simple mathematical summation of amplitudes from SP and SR trials. This indicates extra facilitation beyond what would be accounted for by serial neuronal processing and was not observed during static activation.

**Conclusions:**

We conclude that AL cycling activates shared interneurons in convergent reflex pathways from cutaneous inputs innervating the hand and leg. This enhanced activity has functional implications for corrective responses during locomotion and for translation to rehabilitation after neurotrauma.

## Background

During human bipedal locomotion neuronal features of quadrupedal coordination between arms and legs are likely conserved [1-5]. One method for assessing this coordination is by measuring the modulation of segmental reflexes. For example, after stimulation of nerves in the hand and foot, interlimb cutaneous reflexes in legs and arms are phase-modulated during the walking cycle [6] and during arm and leg cycling [7]. Segmental central pattern generators (CPGs) have been implicated in this coupling regulating arm and leg motion [3]. Coordination of arm and leg motion during walking, creeping, and swimming have also been ascribed to coupled CPG activity [1,8].

Interaction between CPGs presumed to contribute to movement of the arms and legs can be estimated through examination of coupling effects during human rhythmic movement. A coupling effect is operationally defined as a measurable effect of limb movement or motor output on background or reflex muscle activity in another limb. For example, rhythmic arm cycling alters lumbar spinal cord excitability [9] in a manner that interacts with cutaneous inputs from the hand [10,11]. Reciprocal and robust effects arising from leg cycling are also found in arm muscles [12,13].

Recently, effects of rhythmic arm activity on background and reflex motor output have been examined during tasks in which both the arms and legs were rhythmically active such as arm and leg cycling [11,14] and arm and leg stepping [15]. These paradigms can be used as surrogates for “reduced” walking in order to separate arm and leg movement and thus estimate the contributions of the arms and legs to locomotor output.

Functional propriospinal interlimb connections between the fore and hindlimbs have been clearly demonstrated in the cat and in the neonatal rat [16,17], confirming interplay between the cervical and lumbar pattern generators in coordinating rhythmic movement of all four limbs. These observations suggest neuronal circuits regulating the movement of all four limbs are akin to coupled locomotor oscillators. Complementary experiments have also been conducted in the human [7,18].

An important factor related to effective and relevant arm and leg coordination is relaying movement related somatosensory feedback from the hands and feet to the legs and arms and vice versa [3]. Over a century ago, Sir Charles Sherrington made passing mention of these effects in the form of the ‘hand foot’ reflex in the cat [19,20]. Later, Lloyd described a kind of potentiation of interlimb reflexes from the cervical cord to the lumbar cord in the cat [21]. In his experiments an interaction was observed wherein cervical or lumbar nerve stimulation produced potentiated responses in the opposite pathways. For example, cervical stimulation preceding lumbar stimulation produced larger lumbar responses than those arising from cervical stimulation obtained in isolation.

It remains to be determined if a potentiating effect can be induced reliably in humans during locomotor behaviours. Widespread interlimb reflexes evoked in leg muscles by cutaneous stimulation of the hand are phase-modulated and produce functionally relevant changes in ankle trajectory [6]. These reflexes are complementary to the segmental responses evoked by stimulation at the ankle. Despite some differences in the expression of reflex amplitude based upon site of nerve stimulation, there are some common features as well, suggesting the possibility of shared pathways [3]. Currently, little is known about possible convergence or integration of shared reflex systems from interlimb cutaneous networks during locomotion in humans.

The purpose of this study was to investigate convergent reflex effects following cutaneous stimulation of the hand and foot during arm and leg cycling (A&L) by using spatial facilitation. We tested the hypothesis that integration in reflex pathways arising from cutaneous inputs in the hand and foot converge on common interneurons presynaptic to the alphamotoneuronal pool for leg muscles during locomotor activation. Convergence in common interneuronal pathways would be revealed by non-linear summation of reflex amplitudes during simultaneous stimulation of the cutaneous fields in the hand and foot and is predicted to occur only during locomotor activity when CPG elements could make contributions. This outcome would support the assumption that the human locomotor system is organized in a similar fashion to other animals such as the quadrupedal cat.

## Methods

### Subjects and Protocol

**Figure 1:**
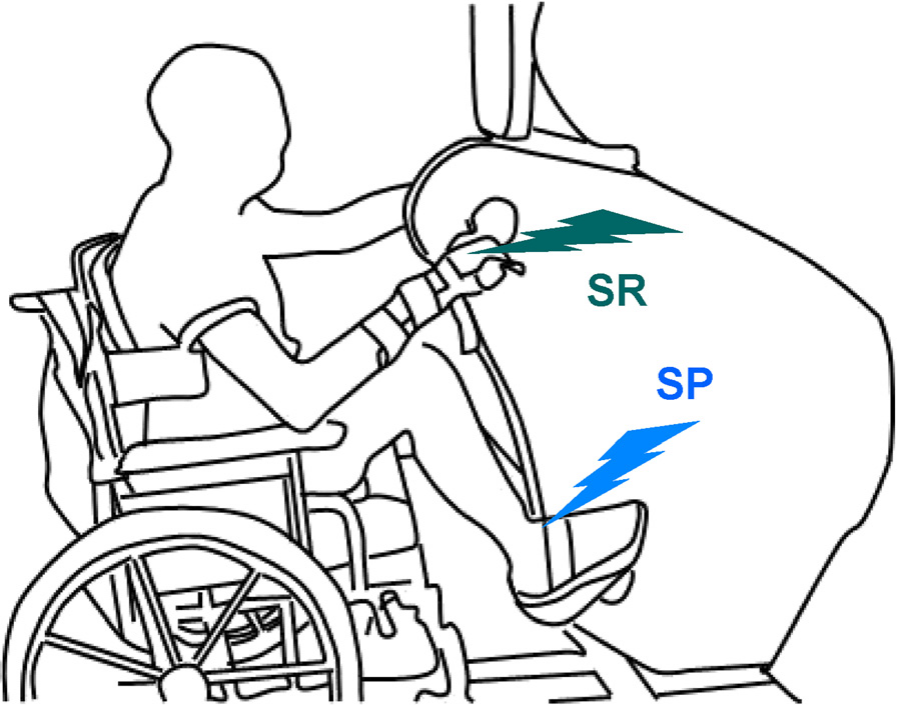
**Experimental set-up for arm and leg cycling (A&L) on the ergometer.** Hand and foot stimulation are indicated by lightning bolts. Abbreviations: SR, superficial radial nerve; SP, superficial peroneal nerve.

Ten healthy subjects (22–49 y), free from neurological or metabolic disorders, participated with informed, written consent according to a protocol approved by the Human Research Ethics board at the University of Victoria. Participants performed seated A&L cycling using an ergometer (SCI FIT PRO 2; see Figure 1) in which the cranks were mechanically coupled to maintain a constant phase relation between arm and leg movement. This mechanical coupling maintained the arms and legs 90 degrees out-of-phase with one another in an orientation akin to upright crawling [1,14]. Additionally, stationary trials involving static activation (~0.5-10% EMG max) of the target muscle VL were performed. The stationary trials were performed while on the ergometer and in the same arm and leg positions as experienced during movement.

A&L cycling was performed in a clockwise direction at a comfortable pace (~1 Hz) and using a low load (~50 W) for approximately 6–8 minutes for each cycling task. This rate is similar to that used previously and is equivalent to a typical walking cadence [1].

A&L cycling was performed in 3 randomly-ordered cutaneous nerve stimulation conditions: 1) input from the hand (superficial radial nerve (SR)); 2) input from the foot (superficial peroneal nerve (SP)); and, 3) combined stimulation from hand and foot (SR + SP).

### Cycle timing and kinematics

Positions of the cranks throughout the movement cycle were obtained from two (i.e., one each for the arms and legs) custom made optical encoders for counting the sprocket teeth and which were mounted to the interior frame of the ergometer. The movement cycle was divided into twelve phases, equivalent to a clock-face with 12 o’clock at the top center position, as described previously [1,14] (see inset Figure 1). Phases of movement are referenced to the position of the legs.

### Muscle activity

Muscle activity (electromyography; EMG) was recorded simultaneously from flexor and extensor muscles in the arms and legs. Knee extensor vastus lateralis (VL) was the target muscle. However, additional muscles studied in order to gauge heteronymous activity included the ankle extensor medial gastrocnemius (MG), ankle flexor tibialis anterior (TA), knee flexor/hip extensor biceps femoris (BF), shoulder flexor anterior (AD) and posterior (PD), and elbow flexor biceps (BB) and extensor triceps brachii (TB)).

EMG signals were amplified (GRASS P511, AstroMed) and bandpass filtered from 100–300 Hz. The processed output was sent to the A/D interface (National Instruments, Austin, TX) and then on to the microcomputer. During the static condition subjects were asked to perform weak knee extension with similar background EMG level measured during AL cycling (0.5-10% of EMGmax).

### Nerve stimulation

In separate cycling trials cutaneous SR or SP nerves were stimulated separately and in combination (SR + SP). A GRASS S88 stimulator with SIU5 stimulus isolation and CCU1 constant current units (AstroMed-Grass Inc.) was used to deliver stimulation in trains of 5 × 1.0 ms pulses at 300 Hz. The radiating thresholds (RT; defined as a clear radiating paresthesia into the innervated skinfield for each nerve), were determined in all subjects. Non-noxious stimulation intensities were adjusted for each subject (SR: ~3.0-4.0 × RT, SP: ~2.0-3.0 × RT). For each stimulus condition, full-wave rectified EMG signals in VL were averaged (n = 20 sweeps).

Stimuli were applied at the onset of the VL EMG burst (corresponding to the onset of the power phase from flexion to extension at ~9-10 o’clock) [1,14]. Stationary trials were performed with the limbs in the same position as used during cycling.

### Quantification of reflexes

Evoked EMG in VL was analyzed for phasic amplitudes and latencies. During offline digital processing using custom written MATLAB (MathWorks Inc.) routines, the stimulus artifact (e.g. from time 0 to ~20 ms post-stimulation) was removed and the sweeps were then dual pass filtered with a Butterworth at 40 hz. Reflexes were initially examined at middle (~80-115 ms), and late (~120-135 ms) latencies. Full analysis was conducted on the late latency only. For each subject, the peak response within the late latency window was determined and a 10 ms average centred around this peak was calculated. This was compared to the unstimulated control data averaged over the same period for statistical purposes. These data from each subject were then averaged across all subjects to obtain the group data.

### Analysis

In all instances, analysis was conducted on averaged values for each subject. Significant differences between conditions was assessed with repeated measures (RM) analysis of variance [One-way RM ANOVA: 4 conditions (SR alone, SP alone, SR + SP and mathematical sum of SP and SR)] in each tasks (cycling and static tasks) and Bonferroni Post-Hoc test using SPSS software Ver.11 (SPSS, Chicago, USA). The F-values and degrees of freedom were obtained after Greenhouse-Geisser correction when appropriate. Descriptive statistics included means ±SEM and statistical significance was set at p</=0.05.

## Results

**Figure 2:**
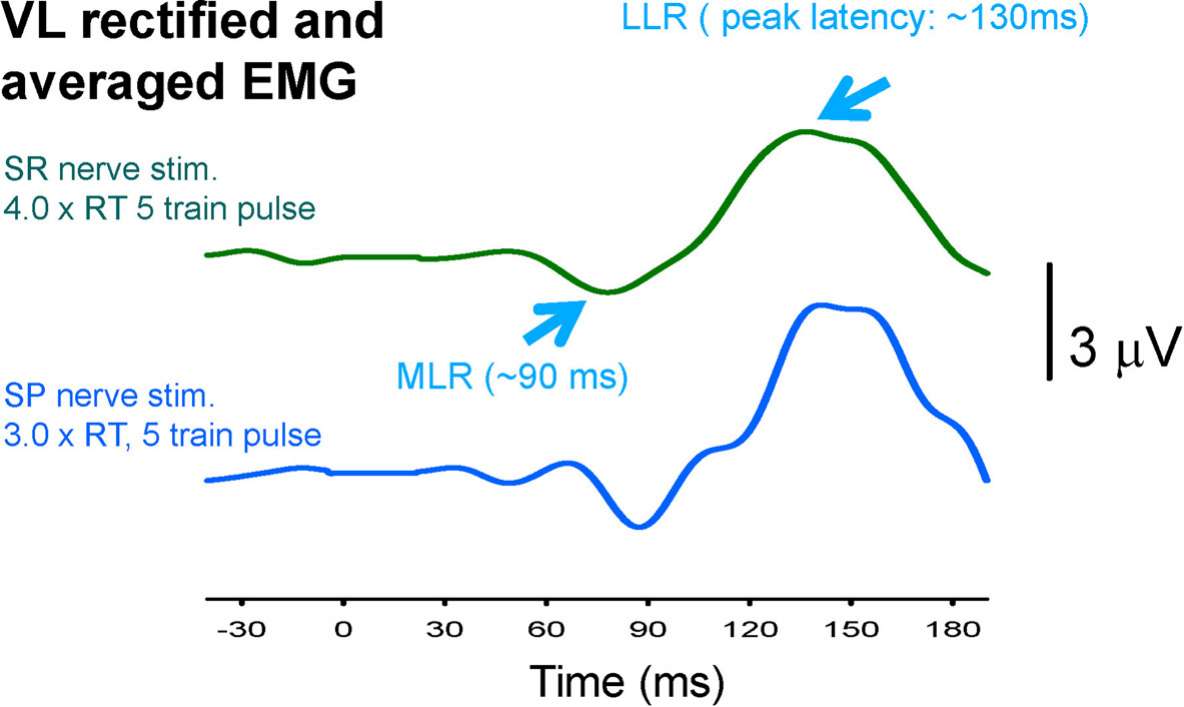
**Example reflex traces evoked by SR or SP nerve stimulation during static activation of VL muscle from a single subject.** Note similar timing of MLR and LLR after both SR and SP stimulation. Stimulus artifacts (e.g. from 0 to ~25 ms) have been removed from both traces and replaced with flat lines. Abbreviations: SR, superficial radial nerve; SP, superficial peroneal nerve; MLR, middle latency cutaneous reflex; LLR, long latency cutaneous reflex.

Stimulation of either SP or SR nerves produced clear cutaneous reflexes in VL muscle during stationary contraction. Sample data from a single subject are shown in Figure 2. Note that responses are of equivalent background level (~10% EMGmax) but are offset for SR (top trace) and SP (bottom trace) in order to see both reflexes clearly. There is considerable similarity in middle (MLR) and late latency (LLR) reflexes as indicated by the arrows.

**Figure 3:**
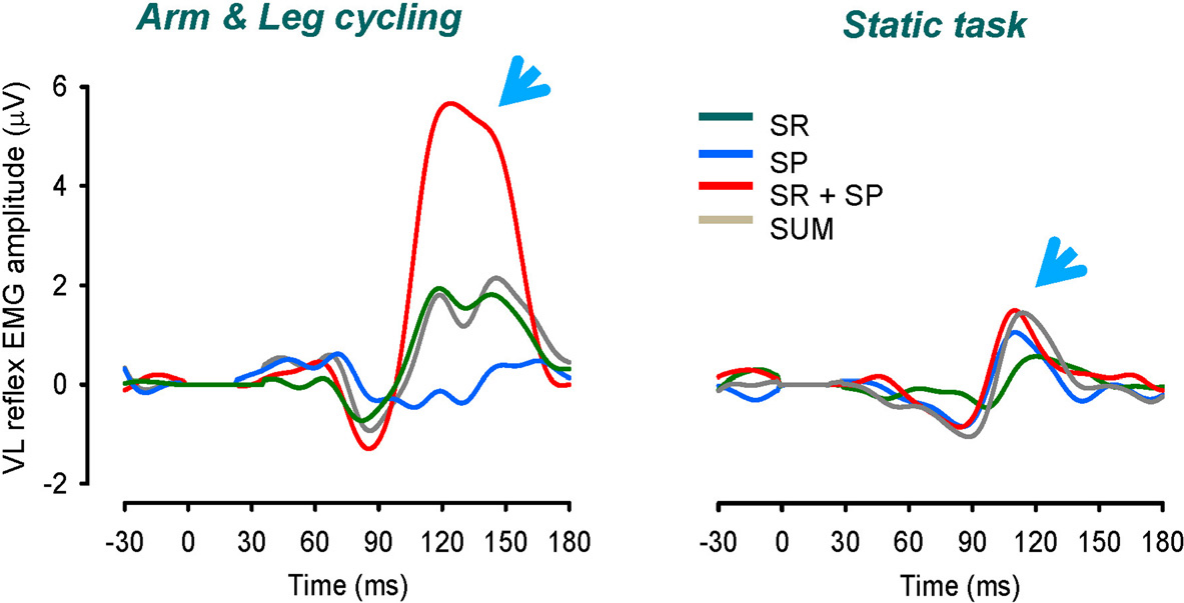
**Example reflexes from a single subject for all experimental conditions.** Reflexes evoked by simultaneous SR + SP stimulation during locomotor activation (arm & leg cycling) show clear amplification (see arrow at left). This effect is absent during static contraction (see arrow at right). Abbreviations: SR, superficial radial nerve; SP, superficial peroneal nerve; SR + SP, simultaneous combined stimulation of SR and SP nerves; SUM, mathematical summation of independent SR and SP reflexes.

Figure 3 illustrates responses in VL during A&L cycling (left panel) contrasted with those during static contraction (right panel) across the 3 stimulation conditions of SR, SP, and simultaneous SR + SP. For reference the simple mathematical sum of reflex traces from SR and SP are shown as “SUM”. There are clear MLR and LLR in all traces. However, the main response of interest is the LLR. The left panel of Figure 3 shows amplified LLR reflex amplitudes with simultaneous SR + SP stimulation (red sweep; note arrow head) during A&L cycling. This amplification was not present during static activation of the target muscle VL (right panel).

**Figure 4:**
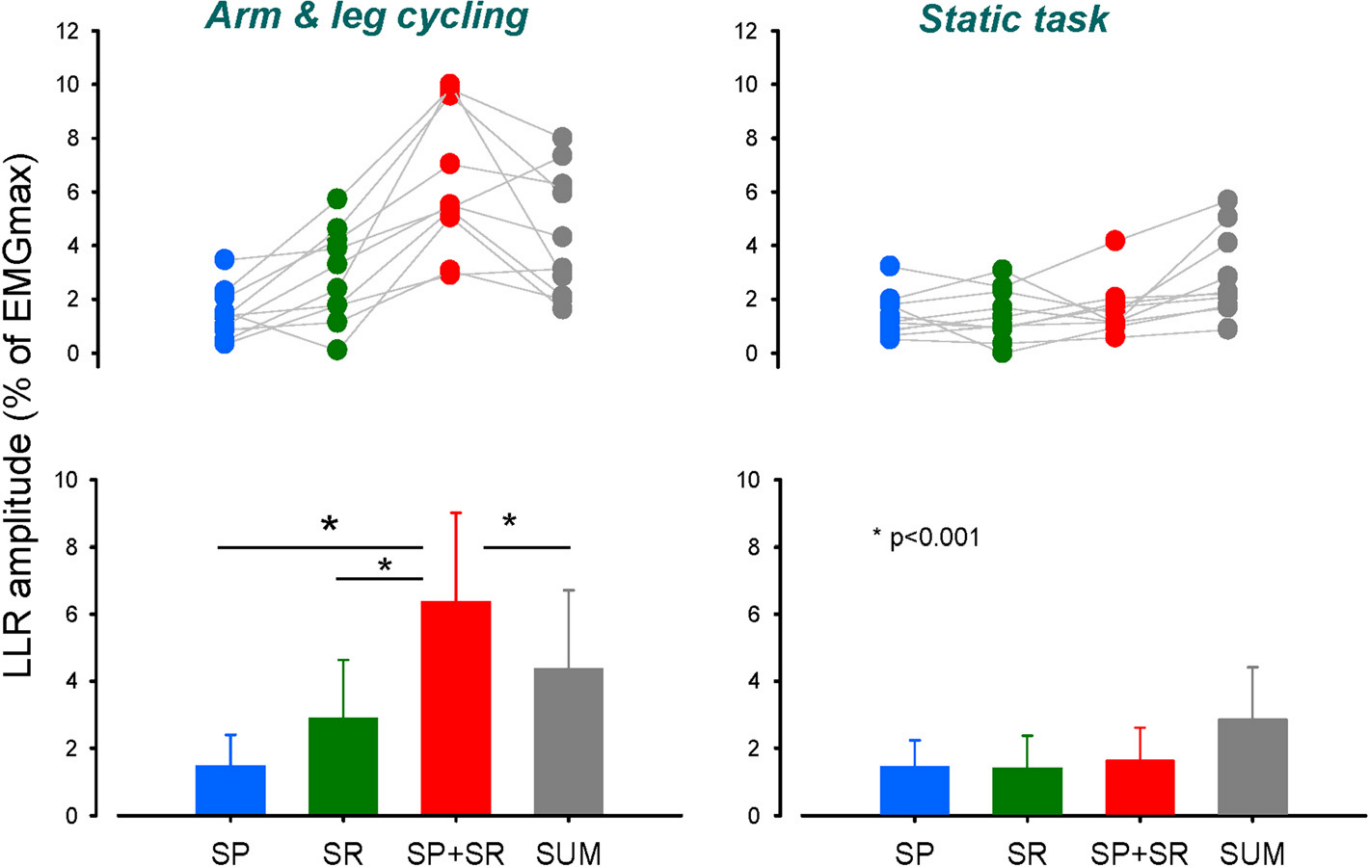
**Simultaneous stimulation of SR and SP nerves produces significantly amplified LLR in VL muscle during locomotion.** Group data across all subjects for all experimental conditions are shown. Reflexes evoked by simultaneous SR + SP stimulation during locomotor activation (arm & leg cycling) are significantly larger than those from independent SR or SP stimulation or the simple summation of SR and SP delivered independently. Abbreviations: SR, superficial radial nerve; SP, superficial peroneal nerve; SR + SP, simultaneous combined stimulation of SR and SP nerves; SUM, mathematical summation of independent SR and SP reflexes.

The amplification of LLR in VL is also evident in the plots for group data. Figure 4 shows data for individual subjects (top plots) along with group averages (bottom plots) during cycling (left panels) and static activation (right panels). The key observations are contained in the group data plot during A&L found at bottom left of Figure 4. Note that amplitudes of LLR with SP and SR stimulation were significantly smaller (p < 0.01) than those obtained for simultaneous stimulation of SP + SR. Critically, a non-linear integration of the inputs from SR and SP nerve stimulation was revealed by the significantly (p < 0.01) larger facilitation of LLR than could be accounted for by simple mathematical summation. During the static task (Figure 4, right panels) there were no differences between VL LLR amplitudes in the SR, SP, and SR + SP stimulation conditions (p > 0.05).

During the static and cycling tasks VL EMG levels were consistently maintained. There were no differences between VL background EMG amplitudes in the SR, SP, and SR + SP stimulation conditions (p > 0.05).

## Discussion

The main result from this study is that the amplitudes of long latency (~120-150 ms) facilitatory cutaneous reflexes are amplified when inputs from the hand (SR n) and foot (SP n) are delivered simultaneously during locomotor activation. This conclusion is based on the empirical observation that reflexes evoked by simultaneous SR + SP stimulation were significantly larger than those for SP or SR stimulation alone. The facilitation was also significantly larger than the simple mathematical summation of amplitudes from SP and SR trials, indicating extra facilitation. This extra facilitation was only observed during rhythmic locomotor activity.

Thus, our results show potentiation of convergent cutaneous stimulation from the hands and feet onto leg muscles in neurologically intact participants. We suggest that A&L cycling activates shared interneurons in the reflex pathways from cutaneous inputs innervating the hand and foot [3,22-24]. The activity of these common interneurons appears directly related to locomotor activity.

### Neurological framework

These results are generally consistent with the work of Lloyd [21]. He used simultaneous cervical (brachial plexus stimulation) and lumbar (L7) afferent volleys to show amplification of lumbar spinal cord activity in the cat. Our results may suggest a spino-bulbar or brainstem integration of interlimb inputs as speculated by Kagamihara et al. [25]. These results may of course also be influence by other supraspinal substrates including transcortical pathways [26]. We are unable to fully extract the pathway from the methodology applied here.

The non-linear summation of effects from SR and SP when delivered simultaneously speaks to a similar mechanism as in the convergence described by Lundberg [22,27]. Our current approach may represent the analogue of spatial facilitation described earlier and reveals task-dependent regulation in this integration in the human during locomotor behaviours. It also remains possible that non-linear summation could arise from subliminal facilitation of high-threshold motor units during simultaneous stimulation. This could produce a related outcome but is not possible to evaluate with the current methodology.

**Figure 5:**
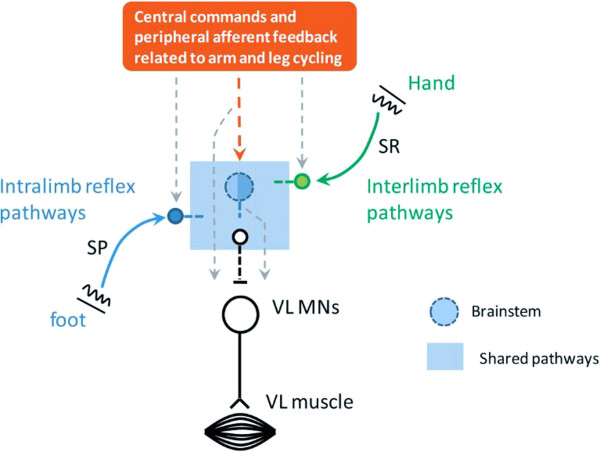
**Schematic diagram outlining a possible neurological framework for integration in cutaneous pathways from the hand and foot during locomotion.** Note that the non-linear amplification of effects from simultaneous cutaneous input from hand (SR) and foot (SP) during arm and leg cycling could occur anywhere within the regions indicated by the shaded square.

A schematic representation of our suggested integration in the human is shown in Figure 5. It is fully admitted that this form of schematic is an oversimplification of the likely set of connections in the human spinal cord and brainstem. However we think it a useful approximation for framing the current observations and future experiments.

The schematic shows cutaneous inputs from the foot, via SP nerve, representing local segmental “intralimb” reflex effects altering excitability of last order lumbar interneurons projecting to motoneurons of the VL muscle. Remote inputs from distant body regions, cutaneous input from the SR nerve in the hand is shown projecting to common last order interneurons sketched by the square, in the form of interlimb reflexes. A key feature in Figure 5 is that the nonlinear summation observed here could arise from convergence in pathways that lie anywhere within the central shaded “square” and which is labelled as “shared pathways”. Our experimental methods do not allow us to effectively delineate the specific locus.

Previous experiments involving human subjects and interlimb reflexes indicate that direct propriospinal networks are involved, at least at short or early latency [28,29]. Here we examined LLR reflexes and the extent to which this integration occurs in supraspinal regions, brainstem, or spinal cord is unknown. However, in light of the observations made by others, particularly Kagamihara and colleagues [25], and the work in reduced animal preparations, [17,30] a brainstem contribution is likely. Hence we have included this as a possible centre for integrating the SR and SP nerve inputs. Our schema is not meant to exclude likely cortical contributions [26] since transmission in afferents arising from arm and leg skin surfaces travel through the cortex with additional projections to the brain stem.

### Functional relevance

Previously we demonstrated interlimb somatosensory linkages in cutaneous pathways between the hands and feet while seated [28], during treadmill walking [6] and during stair climbing [31,32]. This has been extended to conditioning effects of muscle afferent pathways in the legs by stimulation of cutaneous fields in the hand during arm cycling with the legs stationary [10] and during arm and leg cycling [11].

Interestingly, forelimb sensory feedback arising during locomotion has an important regulatory role on hindlimb motor output in the stick insect [33]. Also, during rhythmic leg swing in humans with incomplete spinal cord injury, arm activity has an important function in shaping leg muscle activity [34].

An important consideration of upright bipedal locomotion is a means to integrate coordination between the upper and lower limbs. It has been suggested that interlimb somatosensory networks are well placed to relay information about obstacles and impediments detected by skin surfaces on the hands and feet while walking [2,3,6]. The simplest functional interpretation for the amplification of cutaneous reflexes in leg muscles with simultaneous stimulation of both hand and foot represents the release or priming for stumbling corrections and falling prepartions. In a scenario where the arms and legs encounter obstacles, increased stance support would be required [3]. The enhanced facilitation of knee extensor muscles is directly in line with this prediction. Of course, future experiments are required to more fully elaborate on the characteristics and features of this phenomenon.

### Translational implications for rehabilitation

In a recent paper we presented data on connectivity in arm and leg interlimb cutaneous reflex networks during walking after stroke [35]. We focused on activity in the more affected leg in chronic stroke and examined the modulation of interlimb cutaneous reflexes. Cutaneous reflexes were evoked by stimulation of the contralateral SP nerve (influence from the opposite leg) and superficial radial (influence from the hand) nerves during walking.

Compared to neurologically intact participants, the overall responses in stroke were somewhat blunted. Despite that, somatosensory inputs coming from the arm or opposite leg modulated EMG activity in the more affected leg after stroke. Notably, specific timing of these reflexes at critical parts of the step cycle may improve walking. In that paper we suggested this new information could help advance new therapeutic strategies based on interlimb reflexes and arm and leg interactions. A limitation of this approach, however, is that larger mechanical effects would be of more benefit for gait restoration after stroke. The results of this study indicate that AL cycling activates shared interneurons in the reflex pathways from cutaneous inputs innervating the hand and leg. It is an exciting speculation that integrating our previous observations in stroke with simultaneous stimulation of the hand and foot may show similar non-linear amplification of reflex responses. This observation could enhance development and refinement of novel rehabilitative interventions that make use of intrinsic interlimb pathways to enhance motor ability.

## Conclusions

We conclude that AL cycling activates shared interneurons in convergent reflex pathways from cutaneous inputs innervating the hand and leg. This enhanced activity has functional implications for corrective responses during locomotion and for translation to rehabilitation after neurotrauma.

## Competing interests

The authors declare that they have no competing interests.

## Authors’ contributions

TN was involved in conceptualizing the experiment, conducted the experiments, performed data analysis and edited the MS. TB and TK were involved in experimental design, and data acquisition and analysis. TK edited the original draft and final version of the MS. EPZ was involved in conceptualizing the experiment and wrote an initial draft of the MS and edited the final version. All authors read and approved the final manuscript.
